# Oxford Vaccine Hesitancy Scale (OVHS): a UK-based and US-based online mixed-methods psychometric development and validation study of an instrument to assess vaccine hesitancy

**DOI:** 10.1136/bmjopen-2024-084669

**Published:** 2024-10-09

**Authors:** Jonathan Kantor, Robert C Carlisle, Michael Morrison, Andrew J Pollard, Samantha Vanderslott

**Affiliations:** 1 Oxford Vaccine Group, University of Oxford, Oxford, UK; 2 Department of Engineering Science, University of Oxford, Oxford, UK; 3 Center for Clinical Epidemiology and Biostatistics, University of Pennsylvania Perelman School of Medicine, Philadelphia, PA, USA; 4 HeLEX - Centre for Health, Law and Emerging Technologies, University of Oxford, Oxford, UK

**Keywords:** Public health, Vaccination, PUBLIC HEALTH

## Abstract

**Abstract:**

**Objectives:**

To describe the development, validation and reliability of the Oxford Vaccine Hesitancy Scale (OVHS), a new instrument to assess vaccine hesitancy in the general population.

**Design:**

Cross-sectional validation study.

**Setting:**

Internet-based study with participants in the UK and USA.

**Participants:**

Demographically representative (stratified by age, sex and race) samples from the UK and USA recruited through the Prolific Academic platform.

**Main outcome measures:**

To demonstrate OVHS development, exploratory factor analysis with categorical variables and a polychoric correlation matrix followed by promax oblique rotation on the UK sample was performed. Confirmatory factor analysis with a Satorra-Bentler scaled test statistic evaluating goodness of fit statistics including the root mean squared error of approximation (RMSEA), standardised root mean squared residual (SRMR) and comparative fit index (CFI) was performed on the US sample. Reliability as internal consistency was assessed using McDonald’s omega. Evidence in support of the predictive, convergent and discriminant validity of the scale was assessed using logistic regression ORs of association (OR) or Pearson correlation coefficients.

**Results:**

Data for factor analysis were obtained from 1004 respondents, 504 in the UK and 500 in the USA. A scree plot, minimum average partial correlation analysis and parallel analysis suggested a three-factor 13-item scale with domains of vaccine beliefs (seven items), pain (three items) and personal deliberation (three items). Responses were recorded on a Likert scale ranging from disagree completely to agree completely, with higher score reflecting greater hesitancy. Potential total scores ranged from 13 to 65. Goodness of fit was excellent, with RMSEA=0.044, SRMR=0.041 and CFI=0.977. Predictive validity for COVID-19 vaccination status was excellent, with logistic regression ORs of association (95% CI) of 0.07 (0.04, 0.13), p<0.0001 for the UK sample for each SD increase in OVHS score, suggesting a 93% decrease in the odds of being vaccinated against COVID-19 for each SD increase in OVHS score. Convergent validity between the OVHS score and the 5C short version scale demonstrated a correlation coefficient of 0.32 (p<0.0001). Discriminant validity with an unrelated desire to perform outdoor activities demonstrated an OR (95% CI) of 1.06 (0.88, 1.29), p=0.523 for the UK sample for each SD increase in OVHS score. McDonald’s omega was 0.86 and 0.87 in the UK and US samples, respectively.

**Conclusions:**

The OVHS is a feasible, valid and reliable scale for assessing vaccine hesitancy; further testing is warranted.

**STRENGTHS AND LIMITATIONS OF THIS STUDY**

- Relying on an iterative deductive and inductive approach—including both a detailed review of the extant literature with theoretical models explaining the factors underlying vaccine hesitancy as well as qualitative data—improves our ability to capture relevant domains.
- Including a population of participants who had previously refused vaccination in the early phases of our research further bolsters the generalisability of our findings and incorporate the concerns and priorities of the vaccine-hesitant population.
- Including a large sample of over 1000 participants demographically representative of the UK and US general population further aids in improving the generalisability of our instrument.
- Assessing predictive validity for COVID-19 vaccine refusal provides a real-world practical measure of the utility and performance of the Oxford Vaccine Hesitancy Scale.
- While demographically representative, survey panel approaches have limitations as the population of participants willing to participate in survey panels may not reflect the general population, though this may also be true of the population willing to participate in research studies more generally.

## Introduction

 Vaccine hesitancy is a significant international public health priority, and better appreciating and predicting its aetiology, associations and putative mitigating factors has taken on particular urgency in recent years.^1^,^3^ Indeed, while concerns were raised globally regarding increasing levels of vaccine refusal for preventable childhood illnesses even prior to the COVID-19 pandemic,^4 5^ the ensuing pushback against mandated, or even recommended, vaccination schedules has only increased since vaccine hesitancy was labelled a ‘top-10 threat to public health in 2019,^6 7^ as even primary care physicians admit a significant rate of mistrust in the safety and effectiveness of vaccines^8^ while increasingly ubiquitous social media-heavy diets appear to further exacerbate hesitancy as well.^9^

Given that hundreds of thousands of children and adults are infected with vaccine-preventable diseases each year due to underlying hesitancy,^10^ developing a broader and deeper understanding of the determinants of this phenomenon, as well as a more nuanced appreciation of its manifestations, is a critical task for public health researchers worldwide. While definitional concerns have been raised,^11^ the urgency of the challenge means that tools that could be used by researchers attempting to better tease out the nature of hesitancy are sorely needed.

Several validated scales have been developed previously for evaluating vaccine hesitancy, and a scoping review has evaluated the past decade of scale development in this field.^12^ The Parent Attitudes about Childhood Vaccines (PACV) Scale, the most highly cited scale in evaluating vaccine hesitancy,^11^ was developed to identify parents who are vaccine hesitant.^13 14^ While the PACV has important strengths, including its parsimony and multidimensional approach, it was developed exclusively for childhood vaccinations and fails to capture the rich multidimensionality of contemporary vaccine hesitancy across other groups. The Strategic Advisory Group of Experts on Immunization (SAGE) Vaccine Hesitancy Working Group survey includes three separate sets of items: core questions, Likert-scale questions and open-ended questions.^15^ While these questions address a range of components that align well with the original SAGE definition of vaccine hesitancy, the factor structure of these questions was not explored and psychometric testing was not performed. The Vaccine Confidence Scale (VCS) includes three domains (benefits, harms and trust) over eight items, and the authors found that VCS scores were associated with vaccine refusal, though the modest number of items means that it may fail to capture many of the complex and dynamic contributors to contemporary vaccine hesitancy.^16 17^ The Vaccine Acceptance Instrument, a 20-item scale, examines five domains: safety, effectiveness, acceptance of scheduling, positive beliefs in vaccines and the perceived legitimacy of those mandating vaccinations. Yet the small sample used to develop the scale (246 respondents) coupled with its psychometric limitations (principal component analysis suggested a three-factor solution, which the authors rejected in lieu of their a priori theoretically driven factor structure) means that its generalisability and robustness may be less than ideal. The Vaccine Confidence Index was developed specifically to assess parental trust in the US vaccination system,^18^ though its reliance on an a priori-designated five-factor structure using only eight items suggests the need for multiple factors to load with a single item, likely yielding unstable results. The Vaccine Attitudes Examination scale is a 12-item four-domain instrument that addresses mistrust of vaccine benefits, worries regarding future unforeseen effects, concerns about commercial profiteering and a preference for natural immunity.^19^ However, the use of university students and a non-representative convenience sample for development, and the small number (n=14) of participants included in the domain-generation and item-generation focus groups, may impair the generalisability of this scale. The Vaccine Hesitancy Scale (VHS) was developed by a SAGE working group and included 9 items over two domains: lack of confidence and risks.^20^ Yet in examining only these two inter-related concepts, and focusing exclusively on parental attitudes to childhood vaccination, this scale may not generalise well to other contemporary vaccination scenarios. The 5C scale was developed to assess not only confidence, complacency and convenience but calculation and collective responsibility as well; the authors theorised that if they could evaluate the ‘psychological antecedents to vaccination,’^21^ this would allow them to better understand the complex phenomenon of vaccine hesitancy.^22^ Yet the reliance on an a priori-derived theoretical framework to explain the behavioural antecedents of vaccine hesitancy may limit the ways in which it captures the full range of domains contributing to hesitancy in the real world. Finally, The Multidimensional Vaccine Hesitancy Scale, a 32-item 8-domain scale developed on a survey-panel population, was created in an attempt to broaden the number of domains included in existing scales.^23^ However, it suffers from important limitations including the use of non-representative samples across varying populations, the use of parametric statistics when assessing Likert-scale data, the lack of any trust-related or conspiracy-related items, and significant redundancy, which results in several items that are ‘near synonyms’.^23^

Given the limitations of pre-existing scales, we aimed specifically to practically, feasibly and theoretically improve on existing instruments by developing a scale supported by the practical and theoretical literature on vaccine hesitancy but bolstered by the inclusion of expert opinion and qualitative input from populations of both the general population and the subpopulation of vaccine hesitant individuals. This approach allows us to better capture the range of experiences and expectations associated with the contemporary vaccine hesitancy landscape. By considering a wide array of domains and items and then rigorously applying a robust iterative statistical methodology to domain and item reduction, exploratory and confirmatory factor analysis (CFA), this approach yields a feasible multidimensional scale with significant support for our validity argument.

Opting to forego vaccination may be the result of rational decision-making, an emotional response, mistrust or a combination of factors. The very heterogeneity of the umbrella concept of vaccine hesitancy—as evidenced by those across the ideological spectrum that consider themselves hesitant^24 25^—means that a multidimensional approach to understanding this phenomenon is crucial for public health professionals, behavioural scientists and vaccine researchers. Moreover, quantifying vaccine hesitancy in an accurate, feasible and reproducible fashion may help better tailor future alternative vaccine delivery approaches and technologies to improve the likelihood of vaccine acceptance.^26^ We, therefore, aimed to develop a psychometrically sound, feasible and multidimensional scale to assess the broad concept of vaccine hesitancy based on both the existing literature and qualitative research.

## Methods

### Data collection and population

As part of the predevelopment process, a series of surveys including both open-ended and closed-ended questions were performed via the academic survey site Prolific Academic (Oxford, UK). The first cohort included a convenience sample of 50 respondents, while the second included a separate set of 50 respondents who reported that they had never received any vaccinations against COVID-19.

Oxford Vaccine Hesitancy Scale (OVHS) development was then performed using two distinct assessment cohorts. First, a demographically representative (based on UK Office of National Statistics matched stratification on age, sex, and ethnicity) sample of 500 participants from the UK was enlisted using Prolific Academic. The initial iteration of the scale was administered to these participants via the Qualtrics platform (Provo, Utah, USA). Each participant received a nominal payment of less than £4 for their contribution. The data collected from this UK sample were used for exploratory factor analysis (EFA) to refine the scale’s structure and content.

Subsequently, a second sample consisting of demographically representative (based on 2015 US national census bureau data) US-based respondents was conducted utilising an identical electronic survey recruitment methodology. This second phase of data collection was conducted to perform a CFA, to ensure the model’s robustness across populations, and to assess the scale’s reliability and validity. We elected to perform the CFA on a US sample rather than on a second UK sample to allow us to (a) assess the robustness of the EFA-derived model on a separate population with a different cultural context; (b)highlight the generalisability of our model and (c) efficiently leverage the use of a second population that could both be used to assess model goodness of fit while concomitantly highlighting its generalisability and robustness and avoiding the need for a third sample, consistent with best practices in scale development and our previous work in this area.^27 28^

Each participant’s demographic details were self-declared and coupled with their responses using a unique 25-character alphanumeric identifier to maintain anonymity. The demographic data gathered encompassed a range of attributes, including age, self-identified gender, self-identified race and ethnicity, employment status, educational background, household income and COVID-19 vaccination status.

Our overall methodological approach is similar to our earlier scale development manuscripts.^27 29^ All subjects provided informed consent for participation and were permitted to withdraw from their anonymous surveys at any time. Qualitative analyses were performed using NVivo release V.1.4 (QSR International, Burlington, Massachusetts) and statistical analyses were performed using Stata for Mac version V.16 (Stata Corporation, College Station, Texas).

### Domain and item generation

We approached domain and item generation through an iterative deductive and inductive approach. After a thorough literature review to generate a preliminary domain set, the two 50-member online surveys were used to broaden the range of domains for potential inclusion in the OVHS. Participants also provided feedback regarding sets of test items to gauge their interpretability and were offered the opportunity to note whether any items were difficult to understand, redundant or should be reworded. Qualitative data from open-ended and closed-ended questions, along with feedback from a group of experts from the fields of medicine, public health, vaccinology, social science and engineering science (obtained via workshop group discussions in both the Oxford Vaccine Group and the biology/in vivo cluster meetings of the University of Oxford Department of Engineering Science), contributed to domain development and inclusion to better support face and content validity, an approach consistent with our previous work in this area and established scale development methodology.^27^,^30^

Preliminary responses were recorded using 5-point Likert scale responses and open-ended (free text) responses, as appropriate. Open-ended questions included items such as listing five words that come to mind when thinking about vaccines as well as when thinking about other key stakeholders, such as the people, governments and organisations developing vaccines. These questionnaires also explored the degree to which respondents thought of vaccines as protecting public health and whether they believed vaccines are effective. For the representative samples, responses were recorded on a 5-point Likert scale with options ranging from strongly disagree to strongly agree. Items were reverse scored, as appropriate, ultimately yielding a scale where higher values represent greater levels of vaccine hesitancy.

### Domain and item reduction and exploratory factor analysis

Bartlett’s test of sphericity and the Kaiser-Meyer-Olkin (KMO) measure of sampling adequacy were performed to ensure that the data were amenable for factor analysis.^31^ To establish the ideal number of domains to retain for inclusion in the OVHS, we relied on the minimum average partial correlation for the number of principal components, developing a scree plot and performing parallel analysis for principal components.^30 32 33^ Additionally, item reduction was performed by assessing domain relevance, examining polychoric (item-item) and polyserial (item-test) correlations, culling redundant items and removing those with low communality or low factor loadings.^34^ To ensure that our approach yielded statistically meaningful results with non-continuous data, EFA with categorical variables was performed using polychoric correlations to generate a factor matrix using iterated principal factor analysis followed by promax oblique factor rotation.^35 36^

### Confirmatory factor analysis

Confirmatory maximum likelihood factor analysis using structural equation modelling with a Satorra-Bentler scaled test statistic was performed on the US sample with the model developed in the UK sample to establish the robustness of our model. Goodness of fit indices analysed included the root mean squared error of approximation (RMSEA), the standardised root mean squared residual (SRMR) and the comparative fit index (CFI). Values of RMSEA ≤0.08, SRMR ≤0.08 and CFI ≥0.90 suggest acceptable fit, while values of RMSEA ≤0.05, SRMR ≤0.06 and CFI≥0.95 suggest excellent fit.^37 38^

### Scale reliability and validity

Reliability as internal consistency was assessed using McDonald’s omega, a more robust alternative to Cronbach’s alpha that does not assume equal factor loadings. This was performed for the overall OVHS as well as each subscale.^39^

Predictive validity was assessed by evaluating the association between OVHS score and self-reported vaccination status against COVID-19. Logistic regression ORs of association were assessed per-SD change in OVHS score (the predictor variable) with vaccination status against COVID-19 (with positive vaccination status defined as a self-report of having received at least one vaccination) serving as the dichotomous outcome of interest.

Convergent validity was assessed by evaluating the correlation between the OVHS and the 5C psychological antecedents to vaccination scale (short version), a validated 5-item scale assessing vaccine hesitancy.^22^ Discriminant validity was assessed by examining the logistic regression ORs of association per SD change in the OVHS score predicting agreement with a statement regarding preference for performing outdoor activities. For all analyses, a p value of 0.05 was considered significant.

### Patient and public involvement

We performed this study to develop a scale assessing vaccine hesitancy in the general population. While patient and public involvement was not actively solicited at the initial phase of study design, patients and members of the public were primarily participating as survey respondents. Results will be disseminated and will be used in future studies to assess patient and public preferences regarding vaccination approaches.

## Results

### Respondent characteristics of representative samples

#### UK EFA Sample

For the EFA sample of UK respondents, of the 537 participants who initiated the survey link, 15 declined the survey, 11 timed out (did not complete) and 7 respondents were rejected for failing attention checks. This yielded 504 complete acceptable responses, a 93.9% completion rate. The median (IQR) age was 45 (32, 60), with respondents ranging in age from 19 to 81. Forty-nine per cent of respondents identified as men, and the median (IQR) household income was £40 000 (25 000, 60 000). A total of 14.9% of the participants identified as non-white, and 47.9% reported working full-time.

#### US CFA Sample

For the CFA sample of US respondents, of the 513 participants who initiated the survey link, 10 declined the survey, 2 timed out (did not complete) and 1 respondent was rejected for failing attention checks. This yielded 500 complete responses, a 97.4% completion rate. The median (IQR) age was 46 (31, 60), with respondents ranging in age from 18 to 82. Fifty per cent of respondents identified as men, and the median (IQR) household income was $68 000 (35 500, 110 000). A total of 21.4% of the participants identified as non-white, and 51.6% reported working full-time.

### Domain and item reduction and exploratory factor analysis

We identified 26 potential domains (23 based on literature review and 3 based on qualitative data from the preliminary surveys). Domains included perceived intent of vaccine stakeholders, intentions and willingness, cognition, vaccination behaviour, decision-making and indecisiveness, perception of vaccine benefits, social norms, trust in healthcare professionals, risk assessment, collective responsibility, concerns about pain, perceived profit motives, misinformation, development haste, risks and side effects, inequity, natural immunity preference, personal freedom and control, concern regarding unnecessary vaccinations, vaccination process concerns, past negative experiences, inner conflict, opposition to vaccines, needle fear, complacency and convenience. We assessed a total of 137 items for inclusion in the OVHS after expert consultation, literature review and initial open-ended and closed-ended questionnaire performance. These included positively oriented declarative items such as ‘The benefits of vaccines outweigh their risks’ and ‘Vaccines save lives’, negatively-oriented items such as ‘Getting vaccinated is painful’, and items that address antivaxevangelism such as ‘I would discourage my friends or family from getting vaccinated’. A total of 124 items were culled due to redundancy (58), low domain relevance,^15^ low polychoric or polyserial correlations,^36^ or low communality.^14^ This yielded a final set of 13 items for inclusion in the OVHS.

EFA was performed on the UK sample. With the 13-item EFA, Bartlett’s test of sphericity (p<0.0001) suggested that the data are appropriate for EFA; this was confirmed by the KMO measure of sampling adequacy, which was excellent at 0.90. Individual KMO values ranged from 0.70 to 0.96 for each item. A scree plot, minimum average partial correlation analysis and parallel analysis all suggested a 3-factor solution.^32 40 41^

These three factors reflect domains of (1) vaccine beliefs (7 items), (2) vaccine-induced pain (3 items) and (3) a desire to engage in personal deliberation (3 items). The Eigenvalues for these factors were 6.10, 1.54 and 1.07 accounting for 70.07%, 17.71% and 12.23% of the variance, respectively. Rotated factor loadings are included in table 1. Factor intercorrelations are presented in table 2, and the full OVHS, including scoring key, is outlined in table 3.

**Table 1:** Rotated factor loadings for the OVHS

	Beliefs	Pain	Deliberation
I would vaccinate a child in my care according to the standard vaccine schedule.	**0.9251**	−0.0188	−0.0462
The benefits of vaccines outweigh their risks.	**0.8972**	−0.0630	0.0505
Vaccines save lives.	**0.8806**	0.1093	−0.0830
It is unhealthy for children to be vaccinated as much as they are today.	**0.8720**	−0.0024	0.0705
I believe getting vaccinated is a social responsibility.	**0.8582**	−0.0565	0.0276
I would discourage my friends or family from getting vaccinated.	**0.7707**	0.1557	−0.0121
Drug companies prioritise profit over safety in vaccine development.	**0.6909**	−0.0742	0.1156
I have delayed getting a vaccine specifically because of the needle.	−0.1221	**0.9379**	0.0470
I have refused a vaccine in the past because of the associated pain.	0.2490	**0.7532**	−0.1195
Getting vaccinated is painful.	0.0237	**0.5617**	0.0873
It is important for me to personally understand the risks and benefits of vaccines before agreeing to be vaccinated.	−0.0045	−0.0877	**0.7909**
When considering vaccination, it is important to weigh the positives and negatives.	0.0005	−0.0152	**0.6941**
Doing my own research on vaccines is important to me.	0.0536	0.1303	**0.6775**

Factor loadings > 0.35 are bolded.

OVHSOxford Vaccine Hesitancy Scale

**Table 2:** Factor intercorrelations

Factor	Beliefs	Pain	Deliberation
**Beliefs**	1		
**Pain**	0.4327	1	
**Deliberation**	0.4192	0.1148	1

**Table 3:** The OVHS

Number	Item	Domain
1	I would vaccinate a child in my care according to the standard vaccine schedule.	Beliefs
2	The benefits of vaccines outweigh their risks.	Beliefs
3	Vaccines save lives.	Beliefs
4	It is unhealthy for children to be vaccinated as much as they are today.	Beliefs
5	I believe getting vaccinated is a social responsibility.	Beliefs
6	I would discourage my friends or family from getting vaccinated.	Beliefs
7	Drug companies prioritise profit over safety in vaccine development.	Beliefs
8	I have delayed getting a vaccine specifically because of the needle.	Pain
9	I have refused a vaccine in the past because of the associated pain.	Pain
10	Getting vaccinated is painful.	Pain
11	It is important for me to personally understand the risks and benefits of vaccines before agreeing to be vaccinated.	Deliberation
12	When considering vaccination, it is important to weigh the positives and negatives.	Deliberation
13	Doing my own research on vaccines is important to me.	Deliberation

All items are scored using a 5-point Likert scale ranging from disagree completely to agree completely. Higher scores reflect increasing hesitancy. Items 1, 2, 3 and 5 are reverse scored. The total score can range from 13 to 65.

OVHSOxford Vaccine Hesitancy Scale

### Confirmatory factor analysis

To further assess the model developed in the EFA, a CFA was performed on the US sample. The confirmatory model created with structural equation modelling and a Satorra-Bentler scaled test statistic of the three-factor 13-item model demonstrated excellent goodness of fit characteristics, with RMSEA=0.044, SRMR=0.041 and CFI=0.977.

The median (IQR) OVHS score was 27^24^,^32^ in the UK sample and 28^24^,^35^ in the US sample (p=0.02). Descriptive statistics for each subscale are reflected in table 4.

**Table 4:** Comparison of scores between UK and US samples

	UK samplemedian (IQR)	US samplemedian (IQR)	P value
Beliefs	11 (8–15)	11 (8–17)	0.93
Pain	4 (3–6)	4 (3–6)	0.02
Deliberation	12 (10–14)	13 (11–14)	<0.001
Overall OVHS	27 (24–33)	28 (24–36)	0.02

The table presents the median and IQR values for responses in the domains of Beliefs, Pain, Deliberation and overall Oxford Vaccine Hesitancy Scale (OVHS) score. P values are derived from the Mann-Whitney U test, assessing the statistical significance of differences in the distributions between the two independent samples. P<0.05 suggests a statistically significant difference.

### Scale reliability and validity

Reliability as internal consistency was measured using McDonald’s omega; values for the overall scale were very good at 0.86 and 0.87 in the UK and US samples, respectively. Values for the three subscales are included in table 5.

**Table 5:** Subscale and overall reliability (assessed using McDonald’s omega).

	UK sample	US sample
Beliefs	0.92	0.93
Pain	0.67	0.74
Deliberation	0.71	0.75
**Overall OVHS**	**0.86**	**0.87**

OVHSOxford Vaccine Hesitancy Scale

Predictive validity between the OVHS score and binary COVID-19 vaccination status highlighted the clinical value of the scale, with logistic regression ORs of association (95% CI) of 0.07 (0.04, 0.13), p<0.0001 for the UK sample and 0.24 (0.17, 0.32), p<0.0001 for the US sample, for each SD increase in OVHS score. That is, for each SD increase in OVHS score, there was a 93% reduction in the odds of being vaccinated against COVID-19 in the UK sample and a 76% reduction in the odds of being vaccinated in the US sample.

Convergent validity between the OVHS score and the 5C short version scale demonstrated a correlation coefficient of 0.32 (p<0.0001), further bolstering our validity argument.^22^

Discriminant validity between an unrelated concept—enjoyment of outdoor activities—and OVHS score supported that the OVHS does not predict this unrelated outcome, with logistic regression ORs of association (95% CI) of 1.06 (0.88, 1.29), p=0.523 for the UK sample and 1.14 (0.93, 1.40), p=0.200 for the US sample for each SD increase in OVHS score.

## Discussion

Our work highlights the development and validation of the OVHS, a multidimensional instrument for assessing and quantifying vaccine hesitancy. Ultimately, despite the wide range of domains that were considered, we included only three broad top-level domains in the OVHS: (1) vaccine beliefs, encompassing concepts of efficacy, risk-benefit trade-off, planned behaviour, social responsibility, vaccine evangelism and profiteering; (2) pain, reflecting the prominence of concerns regarding needle-induced pain in driving vaccine attitudes^27^ and (3) deliberation, or the concept that it is incumbent on each individual to do their own research on vaccines, and that this personal deliberative process may contribute to vaccine acceptance or hesitancy.

### Vaccine hesitancy

Vaccine hesitancy has shifted from an area of niche academic and scientific interest to not only a top-10 public health priority but also a socially trending public phenomenon as well.^42 43^ Indeed, while vaccine hesitancy is a multifactorial process, ‘a motivational state of being conflicted about, or opposed to, getting vaccinated… includ(ing) intentions and willingness’,^44^ a recent systematic review of 422 studies addressing vaccine hesitancy highlighted the complexity of the concept, noting that three central approaches to understanding the phenomenon are reflected in the literature: cognition or affect, behaviour and decision-making. The authors suggested that the central theme of vaccine hesitancy is a ‘state of indecisiveness’ regarding the decision to vaccinate.^11^ This model—a state of being conflicted or indecisive regarding vaccination with the process driven by cognition and manifested as decision-making—aligns broadly with the two OVHS domains of beliefs and deliberation.

### Advantages of the OVHS

Several vaccine hesitancy scales have been created recently, but as highlighted previously,^22^ most focus exclusively on a narrow range of domains, and those that take a more multidimensional approach often rely on a priori-developed theoretical models—rather than qualitative research—in developing a range of domains for inclusion.^17^ Still other scales were designed for paediatric vaccines exclusively^13 14 20 45^ and are therefore less appropriate for understanding responses to vaccination more generally.

Indeed, two of our three domains—pain and deliberation—represent a departure from prior scales and may provide additional actionable insight into the drivers of vaccine hesitancy. Understanding vaccine refusal is certainly helpful, but better appreciating if groups or individuals are on a path that may lead towards vaccine hesitancy at an earlier phase has critical public health implications and has not been previously explored. By including a domain addressing pain, our goal was to ensure that the OVHS included not only immutable predictors or antecedents of behaviour but also potentially modifiable associations as well. This is particularly important given that scales such as the OVHS may be used not simply to assess public opinion on a longitudinal or cross-sectional basis but also to aid in the development of alternative approaches and technologies for vaccination promotion or delivery.^26 27^ By addressing modifiable risk factors for vaccine hesitancy, such as pain, the OVHS may be particularly well suited to future public health and interventional studies evaluating ways in which vaccine hesitancy can be not only studied and quantified but also ameliorated as well.

Similarly, the deliberation domain addresses concerns regarding contestable science and social context that have proliferated in the past decade, and particularly since the COVID-19 pandemic, making the concept of vaccine hesitancy far more nuanced and heterogeneous than previously appreciated.^46^,^50^ Indeed, deliberation has been highlighted as a precursor to the distrust and dissent that are the hallmarks of vaccine refusal,^51^ and our scale is the first to include this domain that could potentially serve as an early indicator of future vaccine refusal.

### Study strengths

Our study has several important strengths. First, we used a combination of an evidence-based literature review coupled with qualitative research to develop the domains and items of interest. This approach helps maximise our ability to capture domains and items of interest, particularly given the historical reliance on a priori defined theoretical frameworks with prior scales. Second, our early qualitative research relied on both the general population and vaccine hesitant respondents, a unique approach that provided useful insights into priorities and concerns for the potentially marginalised vaccine-hesitant population. Third, our work relied on large demographically representative samples from both the USA and UK, further bolstering our generalisability and the potential clinical utility of the scale. Fourth, we relied on robust statistical approaches developed for Likert-scale ordinal data, such as EFA using a polychoric factor matrix, to mitigate concerns regarding the appropriate statistical approach to Likert-scale data.^52 53^ Fifth, the outstanding predictive validity of the OVHS—a single SD increase in score is associated with a 76% to 93% reduction in the odds of having been vaccinated against COVID-19—strongly bolsters our validity argument by highlighting the real-world association between OVHS score and vaccine refusal, further supported by our convergent validity with the 5C short form scale. Sixth, the baseline factor intercorrelations suggest that the subscales measure similar, but not identical, concepts, further supporting our validity argument. Seventh, baseline descriptive statistics, such as median OVHS subscale scores, were similar in the UK and US populations, though small (and clinically insignificant) absolute differences did reach statistical significance, likely due to the large sample sizes used in our validation cohorts. Nevertheless, future studies evaluating the OVHS in large and varied populations will be helpful. Finally, the OVHS was designed for use in both adult and paediatric populations, and questions were worded to apply to both adults contemplating their own vaccine choices and parents deciding on the best approach for their children to maximise the utility of our scale for future research studies.

### Limitations

This study also has important limitations. First, survey-panel participants may differ in important ways from the general population, though the platform used in our study has been used widely, our large demographically representative samples from both the USA and UK likely mitigate these issues, and the robustness of our findings across populations suggests that our generalisability is indeed adequate. Second, we included unequal numbers of items across domains, with the beliefs domain accounting for 7 of the 13 items in the scale. Given the breadth of this concept, however, which accounts potentially for several second-order domains, and the meaningful conceptual and statistical partitioning of items across domains, this is unlikely to be a major limitation. Third, the reliability coefficients for the pain and deliberation subscales ranged from 0.67 to 0.75; that said, McDonald’s Omega is sensitive to the number of items in each subscale, and indeed for shorter subscales reliability coefficients likely underestimate the true reliability.^54^ Fourth, qualitative data were collected through a series of open-ended and closed-ended survey questions provided to both a convenience sample and a sample composed of respondents that had never been vaccinated against COVID-19; it is possible that in-person interactive focus groups could have provided additional insights into these responses, though an in-person approach may have constrained the number of vaccine-hesitant respondents that could have been feasibly included in the qualitative portion of the study as such subjects are often challenging to recruit.^55^ Finally, while this manuscript presents the development and initial validity evidence and reliability testing for the OVHS, future work may be helpful to better characterise the scale across differing cultural populations, perform test–retest reliability assessments and conduct invariance testing.

## Conclusions

While vaccine hesitancy is a critical priority for public health investigation, its nebulous nature, with repeatedly shifting definitions of the concept and its black box of underlying contributors means that it is also very difficult to study reproducibly. We, therefore, developed the OVHS to address the real-world observed phenomenon of vaccine hesitancy, particularly as it contributes to vaccine refusal, by approaching the challenge pragmatically and relying on not only a theoretical model of hesitancy but also a qualitative data as well. By yielding a simple three-factor model reflecting vaccine beliefs, concerns regarding pain, and the need for a deliberative process, the OVHS hopefully represents a clinically useful instrument with the potential to aid in both cross-sectional and longitudinal assessments of hesitancy. The OVHS may also provide insight into how the myriad contributors to hesitancy interact and affect behaviour while ultimately assisting in developing alternative technological approaches to vaccination that may broaden access to vaccines worldwide.

## Data Availability

Data are available upon reasonable request.
