# Supplementary material for: Oxford Vaccine Hesitancy Scale (OVHS): a UK-based and US-based online mixed-methods psychometric development and validation study of an instrument to assess vaccine hesitancy
Source: BMJ Open. 2024 Oct 9;14(10):e084669. doi: 10.1136/bmjopen-2024-084669 (PMC11474876; doi:10.1136/bmjopen-2024-084669)
Supplement: Supplementary file 1 [file bmjopen-2024-084669.reviewer_comments.pdf]

PEER REVIEW HISTORY

BMJ Open publishes all reviews undertaken for accepted manuscripts. Reviewers are asked to complete a checklist review form and are provided with free text boxes to elaborate on their assessment. These free text comments are reproduced below.

ARTICLE DETAILS

Title (Provisional)

The Oxford Vaccine Hesitancy Scale (OVHS): a UK-based and US-based online mixed-methods psychometric development and validation study of an instrument to assess vaccine hesitancy

Authors

Kantor, Jonathan; Carlisle, Robert C; Morrison, Michael; Pollard, Andrew J; Vanderslott, Samantha

---

VERSION 1 - REVIEW

**Reviewer**	**1**
**Name**	**Garfield, Sara**
**Affiliation**	**University College London, School of Pharmacy**
**Date**	**15-Mar-2024**
**COI**	**None**

The development of the tool follows a multii-stage process that tests many aspects of validity and reliability. Testing in two populations (UK and US) is a definite strength. However, these are both high income countries and testing in middle and low income countries would add greater generalisability.

The first stage of development is described as qualitative on line asynchronmous focus groups. However, from reading the descriptions of these they do not appear to be focus groups as there was no interaction between the participants. In addition some of the questions were likert scale and these would not be produce qualitative data. Open questions with free text may have given some more in depth responses but likely to be much less in depth than an interview or focus group. It is then not clear how the responses were used to generate the questionnaire.

While the study included patients participants, no patient involvement activities were carried out. For example, there was patient involvement in planning, and monitotring the study, and interpreting and disseminating the results.

My recommendations would be to:

1. Describe the first stage more fully and using correct terminology, including how the results were used in the next stage.

2. To add to the limitations, taking into account my above points.

3. To structure the discussion into different subsections to help signpost the reader and ensure all aspects are covered e.g. summary of results, comparison to the literature, strengths and limitations for practice recommendations for practice, recommendations for future research.

**Reviewer**	**2**
**Name**	**Callaghan, Timothy**
**Affiliation**	**Boston University**
**Date**	**08-Apr-2024**
**COI**	**None**

Thank you for the opportunity to review “The Oxford Vaccine Hesitancy Scale (OVHS): A UK-based and US-based online mixed-methods psychometric development and validation study of an instrument to assess vaccine hesitancy” for possible publication in BMJ Open. This manuscript relies on factor analysis in the UK and the US to assess the creation of a new potential vaccine hesitancy scale emphasizing vaccine beliefs, pain, and personal deliberation. The authors determine that the new scale is feasible, valid, and reliable for assessing vaccine hesitancy. This manuscript is well written and deserves consideration for publication after some revision.

To begin, it would be beneficial for the authors to expand on the introduction. Currently, the discussion of past vaccine hesitancy scales and their limitations is quite limited in the introduction but expansive in the discussion section. I would flip this. Spend more time in the introduction section on past vaccine hesitancy scales so that the discussion can instead be devoted to your new scale. Equally important, more needs to be done in the introduction to justify the need for the new scale. The current introduction makes it clear why your scale is different from past scales, but it needs to do more to emphasize why your scale is an improvement and why scholars should be using your scale moving forward. As a more minor point, I found it surprising that the introduction had very few references to COVID-19 even as it has served to expand vaccine hesitancy in many countries globally.

Next, several changes would be beneficial in the methods and results. First, the authors note that they conducted ‘asynchronous focus groups.’ This method needs considerably more detail provided. Focus groups typically have 8-12 participants each, but you note 50 – how did this work? In addition, how did you conduct these focus groups asynchronously? Second, you note the involvement of a group of experts in your study but their role, the size of the group, how they participated, and their impact are all left too vague. Third, the authors

should do more to justify why EFA was conducted in the UK while CFA was conducted in the US. Wouldn't it make more sense to do CFA in the UK as well before trying to validate in additional countries?

Beyond these issues, I'd encourage the authors to include sample scale items in the text of the manuscript itself. In addition, the drop from 137 items considered for inclusion down to the 13 used seems like a huge drop without much detail. You note that they were culled due to several factors but adding information on this culling process could prove helpful to interested readers and help with reproducibility.

By making these changes, I believe that this manuscript is poised to help improve the study of vaccine hesitancy through the addition of their new scale.

Reviewer 3

Name Heumann, Christian

Affiliation Ludwig-Maximilians-Universitat Munchen, Faculty of Mathematics, Informatics and Statistics, Department of Statistics

Date 03-Jun-2024

COI I have no competing interests.

I congratulate the authors to their well written paper.

I have only a small proposal for improvement. The whole study (including focus groups, UK and US samples with sample sizes), drop outs, etc. could be presented as a flow chart.

VERSION 1 - AUTHOR RESPONSE

1 July 2024

Dear Dr Reeves

Thank you again for considering our work for publication. Please see below for detailed point-by-point responses to the reviewer comments. Our responses are in a bolded font, and citations from our revised manuscript are indented. Highlighting was used to signify added or moved text in the manuscript.

We thank you and the reviewers for their thoughtful and insightful comments and feel that the manuscript is now significantly strengthened thanks to this feedback.

Sincerely,Jonathan Kantor

Reviewer: 1

Dr. Sara Garfield, University College London

Comments to the Author:

The development of the tool follows a multii-stage process that tests many aspects of validity and reliability. Testing in two populations (UK and US) is a definite strength. However, these are both high income countries and testing in middle and low income countries would add greater generalisability.

The first stage of development is described as qualitative on line asynchronmous focus groups. However, from reading the descriptions of these they do not appear to be focus groups as there was no interaction between the participants. In addition some of the questions were likert scale and these would not be produce qualitative data. Open questions with free text may have given some more in depth responses but likely to be much less in depth than an interview or focus group. It is then not clear how the responses were used to generate the questionnaire.

While the study included patients participants, no patient involvement activities were carried out. For example, there was patient involvement in planning, and monitotring the study, and interpreting and disseminating the results.

My recommendations would be to:

1. Describe the first stage more fully and using correct terminology, including how the results were used in the next stage.

We thank the reviewer for their thoughtful and careful comments. Indeed, the description of the qualitative portion of the data collection would be better characterised as a questionnaire survey including a collection of open- and closed-ended questions, given that participants were not able to interact directly. As such, we have modified our descriptions as follows:

As part of the pre-development process, a series of online asynchronous focus groups surveys including both open- and closed-ended questions were performed via the academic survey site Prolific Academic (Oxford, UK).

Following the reviewer's suggestion, we also expanded on some of the specific questions asked:

Open-ended questions included items such as listing five words that come to mind when thinking about vaccines, as well as when thinking about other key stakeholders, such as the people, governments, and organisations developing vaccines. These questionnaires also explored the degree to which respondents thought of vaccines as protecting public health and whether they believed vaccines are effective.

Finally, we appreciate the reviewer's suggestion regarding testing in middle- and low-income countries; we agree that further validation studies would be very helpful and highlight this in our final limitation, noting that 'future work may be helpful to better characterize the scale across differing cultural populations'.

2. To add to the limitations, taking into account my above points.

We again thank the reviewer for this important consideration. We have added a limitation regarding the way in which qualitative data was collected:

Fourth, qualitative data were collected through a series of open- and closed-ended survey questions provided to both a convenience sample and a sample composed of respondents that had never been vaccinated against COVID-19; it is possible that in-person interactive focus groups could have provided additional insights into these responses, though an in-person approach may have constrained the number of vaccine hesitant respondents that could have been feasibly included in the qualitative portion of the study as such subjects are often challenging to recruit.53

3. To structure the discussion into different subsections to help signpost the reader and ensure all aspects are covered e.g. summary of results, comparison to the literature, strengths and limitations for practice recommendations for practice, recommendations for future research.

We again thank the reviewer for this thoughtful and practical suggestion. We have added subsections to our Discussion section to better signpost the reader as follows: vaccine hesitancy, advantages of the OVHS, study strengths, limitations, and conclusions.

Reviewer: 2

Dr. Timothy Callaghan, Boston University

Comments to the Author:

Thank you for the opportunity to review “The Oxford Vaccine Hesitancy Scale (OVHS): A UK-based and US-based online mixed-methods psychometric development and validation study of an instrument to assess vaccine hesitancy” for possible publication in BMJ Open. This manuscript relies on factor analysis in the UK and the US to assess the creation of a new potential vaccine hesitancy scale emphasizing vaccine beliefs, pain, and personal deliberation. The authors determine that the new scale is feasible, valid, and reliable for assessing vaccine hesitancy. This manuscript is well written and deserves consideration for publication after some revision.

To begin, it would be beneficial for the authors to expand on the introduction. Currently, the discussion of past vaccine hesitancy scales and their limitations is quite limited in the introduction but expansive in the discussion section. I would flip this. Spend more time in the introduction section on past vaccine hesitancy scales so that the discussion can instead be devoted to your new scale. Equally important, more needs to be done in the introduction to justify the need for the new scale. The current introduction makes it clear why your scale is different from past scales, but it needs to do more to emphasize why your scale is an improvement and why scholars should be using your scale moving forward. As a more minor point, I found it surprising that the introduction had very few references to COVID-19 even as it has served to expand vaccine hesitancy in many countries globally.

We thank the reviewer for these important and helpful suggestions. As suggested, we have a) flipped the location of the extensive discussion of the vaccine hesitancy scale literature so that the lengthy and detailed discussion of pre-existing scales is now included in the introduction; b) moved the discussion of the advantages of our scale to the introduction, as suggested by the reviewer, to better capture why our scale represents a significant departure from other scales that should be considered by researchers; and c) added some additional references to COVID-19 and its effect on vaccine hesitancy to the introduction.

These sections are now in the introduction:

Several validated scales have been developed previously for evaluating vaccine hesitancy, and a scoping review has evaluated the past decade of scale development in this field.(10) The Parent Attitudes about Childhood Vaccines (PACV) scale, the most highly cited scale in evaluating vaccine hesitancy,(9) was developed to identify parents who are vaccine hesitant.(11, 12) While the PACV has important strengths, including its parsimony and multidimensional approach, it was developed exclusively for childhood vaccinations and fails to capture the rich multidimensionality of contemporary vaccine hesitancy across other

groups. The SAGE Vaccine Hesitancy Working Group survey includes three separate sets of items: core questions, Likert-scale questions, and open-ended questions.(13) While these questions address a range of components that align well with the original SAGE definition of vaccine hesitancy, the factor structure of these questions was not explored and psychometric testing was not performed. The Vaccine Confidence Scale (VCS) includes three domains (benefits, harms, and trust) over 8 items, and the authors found that VCS scores were associated with vaccine refusal, though the modest number of items means that it may fail to capture many of the complex and dynamic contributors to contemporary vaccine hesitancy.(14, 15) The Vaccine Acceptance Instrument (VAI), a 20-item scale, examines 5 domains: safety, effectiveness, acceptance of scheduling, positive beliefs in vaccines, and the perceived legitimacy of those mandating vaccinations. Yet the small sample used to develop the scale (246 respondents) coupled with its psychometric limitations (principal components analysis suggested a 3-factor solution which the authors rejected in lieu of their a priori theoretically-driven factor structure) means that its generalizability and robustness may be less than ideal. The Vaccine Confidence Index (VCI) was developed specifically to assess parental trust in the US vaccination system,(16) though its reliance on an a priori-designated 5-factor structure using only 8 items suggests the need for multiple factors to load with a single item, likely yielding unstable results. The Vaccine Attitudes Examination (VAX) scale is a 12-item 4-domain instrument that addresses mistrust of vaccine benefits, worries regarding future unforeseen effects, concerns about commercial profiteering, and a preference for natural immunity.(17) However, the use of university students and a non-representative convenience sample for development, and the small number (n=14) of participants included in the domain- and item-generation focus groups, may impair the generalizability of this scale. The Vaccine Hesitancy Scale (VHS) was developed by a SAGE working group and included 9 items over 2 domains: lack of confidence and risks.(18) Yet in examining only these two interrelated concepts, and focusing exclusively on parental attitudes to childhood vaccination, this scale may not generalize well to other contemporary vaccination scenarios. The 5C scale was developed to assess not only confidence, complacency, and convenience but calculation and collective responsibility as well; the authors theorized that if they could evaluate the 'psychological antecedents to vaccination'(19) this would allow them to better understand the complex phenomenon of vaccine hesitancy.(20) Yet the reliance on an a priori-derived theoretical framework to explain the behavioural antecedents of vaccine hesitancy may limit the ways in which it captures the full range of domains contributing to hesitancy in the real world. Finally, The Multidimensional Vaccine Hesitancy Scale (MVHS), a 32-item 8-domain scale developed on a survey-panel population, was created in an attempt to broaden the number of domains included in existing scales.(21) However, it suffers from important limitations including the use of non-representative samples across varying populations, the use of parametric statistics when assessing Likert-scale data, the lack of any trust- or conspiracy-related items, and significant redundancy which results in several items that are 'near synonyms.'(21)

Given the limitations of pre-existing scales, we aimed specifically to practically, feasibly, and theoretically improve upon existing instruments by developing a scale supported by the practical and theoretical literature on vaccine hesitancy but bolstered by the inclusion of expert opinion and qualitative input from populations of both the general population and the subpopulation of vaccine hesitant individuals. This approach allows us to better capture the range of experiences and expectations associated with the contemporary vaccine hesitancy landscape. By considering a wide array of domains and items and then rigorously applying a robust iterative statistical methodology to domain and item reduction, exploratory, and confirmatory factor analysis, this approach yields a feasible multidimensional scale with significant support for our validity argument.

Additionally, the COVID-19 discussion in the introduction now includes:

Indeed, while concerns were raised globally regarding increasing levels of vaccine refusal for preventable childhood illnesses even prior to the COVID-19 pandemic,(4, 5) the ensuing pushback against mandated, or even recommended, vaccination schedules has only increased since vaccine hesitancy was labelled a top-10 threat to public health in 2019,(6, 7) as even primary care physicians admit a significant rate of mistrust in the safety and effectiveness of vaccines(8) while increasingly ubiquitous social media-heavy diets appear to further exacerbate hesitancy as well.(9)

Next, several changes would be beneficial in the methods and results. First, the authors note that they conducted ‘asynchronous focus groups.’ This method needs considerably more detail provided. Focus groups typically have 8-12 participants each, but you note 50 – how did this work? In addition, how did you conduct these focus groups asynchronously? Second, you note the involvement of a group of experts in your study but their role, the size of the group, how they participated, and their impact are all left too vague. Third, the authors should do more to justify why EFA was conducted in the UK while CFA was conducted in the US. Wouldn’t it make more sense to do CFA in the UK as well before trying to validate in additional countries?

We again thank the reviewer for these thoughtful and helpful comments. We have clarified our approach to qualitative data collection as noted above, highlighting that the description of the qualitative portion of the data collection would be better characterised as a questionnaire survey including a collection of open- and closed-ended questions, given that participants were not able to interact directly. As such, we have modified our descriptions as follows:

As part of the pre-development process, a series of online asynchronous focus groups surveys including both open- and closed-ended questions were performed via the academic survey site Prolific Academic (Oxford, UK).

Additionally, we added additional detail regarding the approach to the inclusion of expert opinion in our manuscript:

Qualitative data from open- and closed-ended questions presented to these focus groups, along with feedback from a group of experts from the fields of medicine, public health, vaccinology, social science, and engineering science (obtained via workshop group discussions in both the Oxford Vaccine Group and the biology/ in vivo cluster meetings of the University of Oxford Department of Engineering Science), contributed to domain development and inclusion to better support face and content validity, an approach consistent with our previous work in this area and established scale development methodology.(27-30)

Finally, the reviewer raised a very interesting methodological question regarding whether the CFA should have been performed first in the same population as the EFA, rather than in a separate population. We have added some additional clarification of this approach in the body of the manuscript as follows:

We elected to perform the CFA on a US sample rather than on a second UK sample to allow us to a) assess the robustness of the EFA-derived model on a separate population with a different cultural context; b)highlight the generalizability of our model; and c) efficiently leverage the use of a second population that could both be used to assess model goodness of fit while concomitantly highlighting its generalizability and robustness and avoiding the need for a third sample, consistent with best practices in scale development and our previous work in this area.(27, 28)

Beyond these issues, I'd encourage the authors to include sample scale items in the text of the manuscript itself. In addition, the drop from 137 items considered for inclusion down to the 13 used seems like a huge drop without much detail. You note that they were culled due to several factors but adding information on this culling process could prove helpful to interested readers and help with reproducibility.

By making these changes, I believe that this manuscript is poised to help improve the study of vaccine hesitancy through the addition of their new scale.

We again thank the reviewer for their careful reading of the manuscript and their thoughtful and practical suggestions. We have now included several sample scale items in the text itself as follows:

These included positively oriented declarative items such as ‘The benefits of vaccines outweigh their risks’ and ‘Vaccines save lives’, negatively-oriented items such as ‘Getting vaccinated is painful’, and items that address anti-vaxevangelism such as ‘I would discourage my friends or family from getting vaccinated’.

We have also added more detail on the culling process to provide more granular detail for the interested reader:

Of these, 124 items were culled due to redundancy (58), low domain relevance (15), low polychoric or polyserial correlations (37), or low communality (14). This yielded a final set of 13 items for inclusion in the OVHS.

Reviewer: 3

Dr. Christian Heumann, Ludwig-Maximilians-Universitat Munchen

Comments to the Author:

I congratulate the authors to their well written paper.

I have only a small proposal for improvement. The whole study (including focus groups, UK and US samples with sample sizes), drop outs, etc. could be presented as a flow chart.

We sincerely thank the reviewer for their kind words and this helpful suggestion. We have included a flow chart below, and if the editors would like to include it in the manuscript then we are more than happy to fold one of the tables into the body of the manuscript in order to avoid exceeding the table/ figure limits.

VERSION 2 - REVIEW

Reviewer **3**

Name **Heumann, Christian**

Affiliation **Ludwig-Maximilians-Universitat Munchen, Faculty of Mathematics, Informatics and Statistics, Department of Statistics**

Date **11-Jul-2024**

COI **None.**

If the suggested flow chart is included, it should be better balanced wrt the used font sizes.The titles use a big one, the text a too small one.
